# Antimicrobial Treatment of *Pseudomonas aeruginosa* Severe Sepsis

**DOI:** 10.3390/antibiotics11101432

**Published:** 2022-10-18

**Authors:** Johnny Zakhour, Sima L. Sharara, Joya-Rita Hindy, Sara F. Haddad, Souha S. Kanj

**Affiliations:** 1 Division of Infectious Diseases, American University of Beirut Medical Center, P.O. Box 11-0236, Riad El Solh, Beirut 1107 2020, Lebanon; 2 Department of Internal Medicine, Johns Hopkins University School of Medicine, Baltimore, MD 21287, USA; 3 Department of Internal Medicine, Cleveland Clinic Fairview Hospital, Cleveland, OH 44111, USA; 4 Division of Infectious Diseases, Department of Medicine, College of Medicine, Mayo Clinic, Rochester, MN 55902, USA

**Keywords:** *Pseudomonas aeruginosa*, severe sepsis, antibiotics, antimicrobial resistance, hospital-acquired infections

## Abstract

*Pseudomonas aeruginosa* is a pathogen often encountered in a healthcare setting. It has consistently ranked among the most frequent pathogens seen in nosocomial infections, particularly bloodstream and respiratory tract infections. Aside from having intrinsic resistance to many antibiotics, it rapidly acquires resistance to novel agents. Given the high mortality of pseudomonal infections generally, and pseudomonal sepsis particularly, and with the rise of resistant strains, treatment can be very challenging for the clinician. In this paper, we will review the latest evidence for the optimal treatment of *P. aeruginosa* sepsis caused by susceptible as well as multidrug-resistant strains including the difficult to treat pathogens. We will also discuss the mode of drug infusion, indications for combination therapy, along with the proper dosing and duration of treatment for various conditions with a brief discussion of the use of non-antimicrobial agents.

## 1. Introduction

*Pseudomonas aeruginosa* is the third most common cause of Gram-negative bloodstream infections (BSI) with a mortality rate of up to 30% at 30 days, which surpasses that of *Staphylococcus aureus* and other Gram-negative bacteria causing BSI [1,2,3,4,5]. In neutropenic cancer patients, pseudomonal sepsis is the leading cause of death [6,7]. Furthermore, *P. aeruginosa* is classified as a “critical” pathogen by the World Health Organization (WHO), a “serious threat” by the Center for Disease Control and Prevention (CDC) and was included as one of the “ESKAPE” pathogens causing nosocomial infections worldwide [8]. Aside from its intrinsic resistance to many antimicrobials, acquired resistance makes treatment even more challenging. As resistant strains become more predominant, the risk of inappropriate empiric treatment increases, which results in higher risk of mortality [9,10].

*P. aeruginosa* sepsis is most often encountered in the setting of nosocomial infections in neutropenic patients, critically ill patients, or patients with burn injuries, cystic fibrosis, catheter-associated urinary tract infections (UTIs), surgical site infections, or intra-abdominal infections [11,12]. *P. aeruginosa* is a rare cause of community acquired sepsis except in immunocompromised patients, or patients with structural lung disease [13,14,15]. Given the severity of pseudomonal sepsis and increased antimicrobial resistance (AMR), initial management is often suboptimal. In fact, multidrug resistant *P. aeruginosa* (MDR-PA) has been reported as an independent risk factor for mortality in patients with hospital-acquired pneumonia [16]. Moreover, delayed proper therapy for pseudomonas pneumonia has been shown to significantly increase mortality when compared to appropriate therapy [17]. Therefore, it is essential to identify patients at risk for *P. aeruginosa* generally and MDR-PA particularly, to guide empiric therapy. Patients with invasive devices (indwelling catheters), intensive care unit (ICU) admission, bedridden status, diabetes mellitus, tracheostomy, recent history of treatment with broad-spectrum antimicrobials, history of carbapenem-resistant *P. aeruginosa* (CRPA) infection, burn wounds, pressure ulcers, neutropenia or other immunocompromising conditions should be considered at high risk for MDR-PA sepsis [18,19,20,21]. Moreover, some sites of infection are more likely to result in sepsis than others. For instance, *P. aeruginosa* pneumonia has been associated with the highest risk of sepsis, severe disease course, and mortality [5].

Although some dermatologic findings such as ecthyma gangrenosum, diffuse maculopapular lesions, tender vesicles or pustules in clusters, and areas of cellulitis that may progress to necrosis may be suggestive of pseudomonal sepsis, it is clinically indiscernible from sepsis due to other pathogens [22]. In addition, fever, tachycardia, tachypnea, and hypotension are unspecific signs that accompany most other Gram-negative sepsis syndromes. Hence, in the presence of risk factors for *P. aeruginosa* infection, antipseudomonal empirical antibiotic therapy (EAT) should be quickly initiated to reduce the risk for inadequate initial therapy, a well-established risk factor for increased 30-day mortality [23,24,25,26]. Prompt bacterial identification and susceptibility testing are essential to guide definitive antibiotic therapy. Conventional techniques that rely on bacterial culture often take several days to be reported and increase the risk of inadequate EAT. Novel techniques like whole genome sequencing (WGS), whole metagenomic sequencing (WMS) and matrix-assisted laser desorption/ionization-time of flight mass spectrometry (MALDI-TOF MS) can reduce time to microbial identification and susceptibility testing from days to hours and even minutes. However, high cost, necessity for a database, lack of standardization, and inability to determine the minimum inhibitory concentration (MIC) are limiting the wider use of those techniques [27].

In this article, we will be discussing the latest evidence regarding the management of *P. aeruginosa* sepsis including the selection of antimicrobials for empiric and targeted therapy, as well as key factors such as dosing, duration of treatment, data on combination therapy, and alternative therapies.

## 2. Empirical Antimicrobial Therapy

EAT in sepsis should consider the patient’s allergies, comorbidities, the primary site of infection, prior antibiotic exposure, as well as local susceptibility patterns [20]. AMR should be highly suspected if there is recent admission to a hospital unit where prevalence of MDR-PA is greater than 20% or if the patient has received antipseudomonal beta-lactam antimicrobials within the past three months [28]. Although some studies reported trends towards decreased resistance of *P. aeruginosa* [29], low and middle income countries (LMICs) still suffer from a high burden of AMR [30]. The CDC reports that 32,600 cases of MDR-PA infections occurred in patients hospitalized in the United States in 2017, resulting in 2700 deaths [31]. For *P. aeruginosa*, MDR is defined as resistance to at least one agent in three or more antibiotic classes, extensive drug resistance (XDR) is defined as resistance to at least one agent in all but two or fewer antibiotic classes, and pan-drug resistance (PDR) is non-susceptibility to all agents [32]. Most recently, it was suggested to label MDR-PA as difficult to treat (DTR) when it is resistant to piperacillin-tazobactam, ceftazidime, cefepime, aztreonam, meropenem, imipenem-cilastatin, ciprofloxacin and levofloxacin [33,34].

Combination therapy in *P. aeruginosa* is often used to decrease the risk of inadequate EAT by combining drugs with multiple mechanisms of action. In a recently published multicenter retrospective study including 1017 neutropenic patients with *P. aeruginosa* bacteremic pneumonia, inappropriate EAT was given to 23% of patients and was associated with infection with MDR-PA. Additionally, inappropriate EAT was associated with increased 30-day mortality while appropriate EAT was independently associated with improved survival [10]. No consensus has been reached regarding the use of empirical combination versus monotherapy in *P. aeruginosa* sepsis, mainly due to the lack of robust prospective studies to provide strong levels of evidence [35]. In fact, a prospective study found no differences in outcomes between patients who received empirical combination antibiotics when compared to monotherapy [36]. A meta-analysis that included 1721 patients showed no difference in mortality among patients with pseudomonal infections who were treated empirically with beta-lactam monotherapy or combination therapy with the addition of an aminoglycoside (AG) or a fluoroquinolone (FQ) [37]. Furthermore, a Cochrane review that included 69 randomized controlled trials (RCTs) with a total of 7863 patients comparing beta lactam monotherapy and combination with an AG in the management of sepsis showed no difference in mortality in the *P. aeruginosa* subgroup analysis and significantly increased nephrotoxicity with combination therapy [38]. Moreover, a post hoc analysis of 593 patients with *P. aeruginosa* bacteremia showed no benefit of empiric combination therapy [39] and another meta-analysis of 4980 patients showed no difference in mortality, microbiological, or clinical cure when using empirical combination vs. monotherapy for patients with *P. aeruginosa* BSI or pneumonia [40]. On the other hand, a retrospective cohort study by Micek et al. including 305 patients with *P. aeruginosa* BSI showed that using combination therapy while awaiting for identification and susceptibility testing decreased the risk of inadequate EAT from 79.4% to 65.5% (*p*-value = 0.011). Additionally, mortality was significantly higher in patients who received inappropriate EAT (30.7% versus 17.8%, *p*-value = 0.018). In that study, inappropriate EAT, respiratory failure and septic shock were found to be independent risk factors for in-hospital mortality [41]. A recent meta-analysis of four studies that evaluated all-cause mortality (total of 148 patients), showed a significant decrease in mortality with combination therapy for severe infections caused by *P. aeruginosa* (OR 0.31, 95% CI 0.1–0.97, *p*-value = 0.045) [42].

**Scheme 1:**
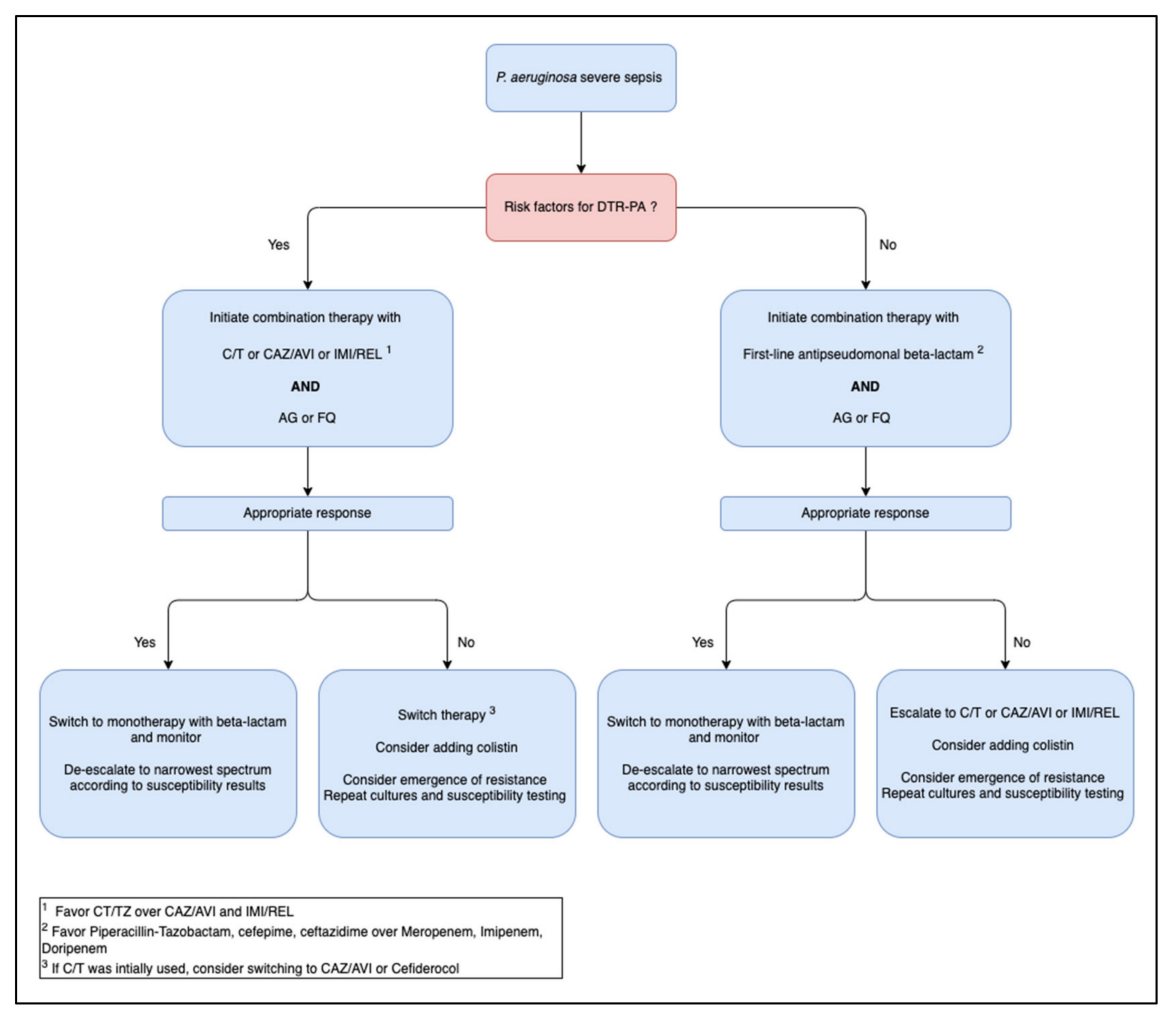
Antimicrobial management of *P. aeruginosa* severe sepsis DTR-PA, difficult to treat *Pseudomonas aeruginosa****;*** C/T, ceftolozane-tazobactam; CAZ/AVI, ceftazidime-avibactam; IMI/REL, imipenem-cilastatin-relebactam; AG, aminoglycoside; FQ, fluoroquinolone.

Given the rise of AMR and the risk of inadequate EAT, combination empiric therapy should be highly considered in cases of severe sepsis [43]. The Surviving Sepsis campaign recommends combination empirical therapy during acute illness [44]. Two different mechanisms of action are preferred, typically a backbone beta-lactam (conventional or novel depending on risk of AMR) combined with an AG or a FQ [18,28]. Although one study suggested better outcomes when FQ was used instead of AG as a second agent [45], the choice of agent should be guided by local susceptibility patterns [28]. A retrospective cross-sectional analysis of blood and respiratory *P. aeruginosa* isolates from patients admitted to the ICU found that the combination with the highest susceptibility was piperacillin-tazobactam combined with an AG, while the combination with the lowest susceptibility was a carbapenem combined with a FQ [46]. Additionally, isolates were found to have less resistance to combinations with AG than those with FQ. A pharmacokinetic/pharmacodynamic (PK/PD) prospective randomized controlled trial suggested that a higher dose of amikacin (25 mg/kg) for patients with severe sepsis and at risk for *P. aeruginosa* infection was more likely to achieve an MIC that is closest to the EUCAST susceptibility breakpoint than standard dosing (15 mg/kg) [47]. Above all, the choice of empiric antimicrobial regimen should consider the potential for co-resistance to multiple first-line agents. For instance, a multinational microbiological study including 1783 isolates of MDR-PA from patients with *P. aeruginosa* BSI reported that co-resistance to many first-line antipseudomonal agents was very common, especially between piperacillin-tazobactam, meropenem and ceftazidime. Among antimicrobials that were included in the study, only Ceftolozane-tazobactam (C/T), a novel beta-lactam-beta-lactamase inhibitor combination, achieved significant additional activity against strains that exhibited resistance to one of the first-line agents [48]. Those findings suggest that C/T may be considered for empirical therapy if local rates of PA resistance to first-line agents is high. If combination empiric therapy is used, we highly recommend prompt de-escalation once there is clinical improvement and susceptibility results are available (Scheme 1). Additionally, although 40% of *P. aeruginosa* BSIs will have an unidentifiable origin, we recommend prompt source control when possible to improve patient outcomes [4,7].

## 3. Targeted Therapy for *P. aeruginosa* Sepsis

### 3.1. P. aeruginosa Sensitive to First Line Antipseudomonal Agents

*P. aeruginosa* is intrinsically resistant to several antibiotics due to the low permeability of its outer membrane, expression of various efflux pumps, and the production of antibiotic-inactivating enzymes such as inducible cephalosporinases. First-line beta-lactam agents for *P. aeruginosa* coverage include beta-lactam/beta-lactamase-inhibitor combinations (BL/BLI) (piperacillin-tazobactam and ticarcillin-clavulanate) and cephalosporins with antipseudomonal activity (ceftazidime, cefepime, and cefoperazone). Cefepime is the most commonly used beta-lactam antibiotic for *P. aeruginosa* [49]. Fluoroquinolones (ciprofloxacin and levofloxacin) remain currently the only oral treatment options for quinolone-sensitive *P. aeruginosa*. However, ciprofloxacin is superior to levofloxacin given the higher risk of emergence of quinolone-resistant *P. aeruginosa* with the use of levofloxacin [50]. Additionally, older FQ are less effective in acidic environments like UTIs [51]. Newer FQ such as finafloxacin and delafloxacin offer more activity in acidic environments but are yet to be widely available [52].

Second line agents for *P. aeruginosa* sepsis are carbapenems, including meropenem, imipenem, and doripenem. Meropenem is often preferred over imipenem given the latter’s higher propensity to induce resistance during treatment [53]. Doripenem was shown to be more active in vitro against *P. aeruginosa* compared to meropenem and imipenem but this has not been proven in clinical studies [54,55]. Nonetheless, cephalosporins should be favored over carbapenems when applicable due to more potent activity and narrower spectrum [56] as well as less propensity to select for future resistance [57].

Other agents include the monobactam class (aztreonam) which can be used as an alternative for patients with penicillin allergy. Gentamicin, tobramycin, and amikacin are all AG that can be active against *P. aeruginosa* but are not indicated as monotherapy except for UTIs, as they are associated with higher mortality rates [58]. In the case of severe sepsis, the pathophysiological shifts may lead to an increased volume of distribution and augmented renal clearance and may lead to suboptimal AG concentrations and potentially poorer outcomes [59]. For optimal coverage, we prefer tobramycin or amikacin over gentamicin [60]. Otherwise, plazomicin, a newer AG, was shown to be less effective and is currently only indicated in the treatment of UTIs [61].

Emergence of resistance during the course of treatment is a serious concern. Such is the case of a cohort of 271 patients with various *P. aeruginosa* infections receiving antipseudomonal antimicrobial therapy, where emergent resistance was reported in up to 10% of cases [57]. Additionally, standard susceptibility testing may not be as accurate in identifying resistance when hospitalization duration increases. This is likely due to development of resistance or acquisition of drug-resistant hospital-acquired strains, especially with prolonged stay in the ICU. Studies have indicated that initial antibiograms become unreliable as a predictor of susceptibility of *P. aeruginosa* after 1–2 weeks of hospitalization, particularly in the ICU, with a significant increase in MIC for multiple anti-pseudomonal agents [62,63]. Among conventional treatment agents, imipenem was the most likely to cause resistance emergence and ceftazidime was the least likely [57].

### 3.2. P. aeruginosa Resistant to First Line Therapy

*P. aeruginosa* can develop resistance through multiple mechanisms including selection of chromosomal mutations or horizontal acquisition of broad-spectrum resistance genes. Many resistance mechanisms are involved and include beta-lactamase production, AG-modifying enzymes, efflux pumps, porin loss, and various target site modifications [56]. Treatment options for CRPA is challenging given the variety of resistance mechanisms like the production of carbapenemases of different classes, outer membrane protein modification (OprD) or efflux pumps (MexAB, MexXY) (Table 1). Novel anti-pseudomonal drugs have been developed in response to this challenge to address the increase in resistance, which has been reported in up to 54% of nosocomial *P. aeruginosa* infections [64,65,66]. These include novel BL-BLI like C/T, ceftazidime/avibactam (CAZ/AVI), and imipenem-cilastatin/relebactam (IMI/REL) or novel cephalosporins like cefiderocol [20].

C/T has potent intrinsic anti-pseudomonal activity owing to its greater affinity to all essential penicillin-binding proteins (PBP) including PBP1b, PBP1c, and PBP3. Based on RCTs, the US Food and Drug association (FDA) and the European Medicines Agency (EMA) have approved the use of C/T in complicated intra-abdominal infections (IAIs), UTIs, and hospital-acquired pneumonia including ventilator-associated pneumonia (VAP) [72]. Subset analysis in the clinical trials showed that patients with *P. aeruginosa* had a favorable outcome compared to carbapenems in the HAP trial [73], carbapenems combined with metronidazole in the complicated IAI trial [74], and levofloxacin in the complicated UTIs trial [75]. CAZ/AVI is a novel BL/BLI combination approved by the FDA and the EMA for treatment of complicated UTIs, IAIs and infections with Gram negative resistant pathogens [76,77]. C/T and CAZ/AVI have been considered key therapeutic agents against resistant *P. aeruginosa* strains. However, since the commercialization of these agents, there has been emergence of resistance of *P. aeruginosa* following therapy, particularly with highly cephalosporin-resistant conferring mutations [78]. Data for treatment associated resistance in novel agents is still inconclusive, but it appears that the highest risk is with C/T and CAZ/AVI with common cross resistance to both agents [79,80]. Currently, real-world data is scarce and does not show superiority of an agent compared to another and therefore the choice of antimicrobial therapy should be based on the susceptibility profile which can vary according to the local epidemiology with regional variability [81]. Since the introduction of C/T, case reports and case series of MDR *P. aeruginosa* infections treated with C/T have demonstrated the clinical efficacy of this formulation, including its use to treat infections in critically ill patients, and those with cystic fibrosis [82,83]. The European Society of Clinical Microbiology and Infectious Diseases (ESCMID) recent guidelines for the treatment of infections caused by MDR Gram-negative bacilli suggest treatment with C/T as the single first choice for severe pseudomonal infections like severe sepsis [35]. As for the Infectious Diseases Society of America (IDSA), their guidelines recommend treatment with either C/T, CAZ/AVI, or imipenem/relebactam for infections with DTR *P. aeruginosa* outside the urinary tract [33]. In fact, although experience with CAZ/AVI in the management of *P. aeruginosa* infections is more limited, adding avibactam to ceftazidime has shown success in lowering MICs of many XDR *P. aeruginosa* isolates [84]. When considering XDR *P. aeruginosa*, an important concept is that although C/T is more likely to be active than CAZ/AVI, there are some C/T-resistant strains that can be susceptible to CAZ/AVI [85]. Therefore, in vitro susceptibilities to both agents should be obtained whenever possible.

Cefiderocol is a novel siderophore cephalosporin that can overcome efflux pumps. The IDSA recommends this agent as an alternative therapy when other novel BL/BLI agents are unavailable or if there is resistance or intolerance. A recent RCT compared the outcomes of patients with infections due to carbapenem-resistant bacteria treated with cefiderocol or best available therapy (BAT) [86]. Although the mortality rate was higher in the cefiderocol arm, the number of patients with *P. aeruginosa* infection was small, and increased mortality was only observed in patients with a mono- or polymicrobial infection including *Acinetobacter baumannii*. Furthermore, the results suggest that cefiderocol performed as well as BAT, but was not associated with significantly decreased mortality or reduced adverse events like what was reported from studies on newer BL/BLIs [87,88]. A recently published study of *P. aeruginosa* isolates resistant to C/T and CAZ/AVI concluded that cefiderocol was the most active agent against these isolates, with only one resistant clinical isolate (R504C substitution in PBP3) [78]. Imipenem-cilastatin/relebactam was also active against all isolates except two that carried the VIM-20 carbapenemase. In the same study, newer combinations such as cefepime/zidebactam and cefepime/taniborbactam displayed activity against most of the isolates, but resistance was observed in some strains with PBP3 amino acid substitutions and those that overexpressed mexAB-oprM or mexXY efflux pumps.

Evidence for the combination IMI/REL is derived from the RESTORE-IMI 1, a randomized controlled phase 3 trial, comparing IMI/REL to a combination of colistin and imipenem for patients with Gram-negative infections of which 77% were due to *P. aeruginosa*. There was a trend of lower mortality in the arm that was treated with the novel agent compared to combination therapy, but a significantly lower rate of adverse effects and nephrotoxicity [87]. In the subgroup analysis, patients with pneumonia as well as those with renal insufficiency had a higher mortality perhaps owing to lower concentration achieved with the given doses (86). As for meropenem/vaborbactam, it is not recommended given that the addition of vaborbactam was not found to restore susceptibility to meropenem-resistant strains [33].

Despite great outcomes associated with novel agents, these therapies remain inactive against most metallo-beta-lactamase-(MBL)-producing *P. aeruginosa* strains. The monobactam aztreonam is unique by demonstrating stability to hydrolysis by MBLs and may maintain activity against MBL-producing *P. aeruginosa* [89]. Many strains of MBL-producing *P. aeruginosa* will also contain mechanisms of resistance against aztreonam, such as increased expression of pseudomonas derived cephalosporinases (PDCs). Nevertheless, aztreonam is an attractive option in combination with CAZ/AVI for the treatment of infections caused by MBL-producing *P. aeruginosa* [90]. Cefiderocol has also shown activity against all carbapenemase classes including MBL but more clinical evidence is needed [91].

As for polymyxins (polymyxin B and colistin), the IDSA’s latest guidelines for the treatment of DTR *P. aeruginosa* recommends against their use when novel options with less nephrotoxicity are available [33]. However, given the increase in resistance rates and scarcity of novel antibiotics in LMICs, colistin has been increasingly used. Studies have shown that colistin can be used as salvage therapy when options are limited [92], and that it can be associated with a lower expected incidence of nephrotoxicity than previously expected. However, renal function should be closely monitored during therapy with appropriate dose adjustments.

Fosfomycin is an interesting choice for DTR *P. aeruginosa* given that it retains activity against some XDR and PDR strains which may be useful especially in critically ill patients with severe sepsis [93]. A case series including 48 critically ill patients, of whom 17 had infection with MDR-PA and 10 with severe sepsis, evaluated the efficacy of intravenous fosfomycin mainly in combination with colistin. Patients who received fosfomycin were found to have an all-cause 28-day mortality of 37.5%. Additionally, adverse events were minor and included nausea and reversible hypokalemia while resistance emergence to fosfomycin was found in only 3 patients [94]. Another retrospective study comparing outcomes between patients with CRPA pneumonia receiving a combination of doripenem and colistin or doripenem and fosfomycin found similar outcomes between both groups; however, results should be cautiously interpreted given the small size of the study’s population (49 patients) [94]. It should be noted that intravenous fosfomycin should not be given as monotherapy except in cases of uncomplicated UTI; otherwise, it should be given in combination with other agents for bacteremia, nosocomial pneumonia and complicated skin and soft tissue infection to avoid emergence of resistance [43].

While definitive combination therapy may exert a synergistic effect and possibly reduce the emergence of resistance, it can also result in increased side effects and unnecessary costs [56]. We have previously discussed the lack of rigorous evidence regarding the efficacy and safety of empirical combination therapy. Similarly, the evidence concerning the efficacy and safety of definitive combination antimicrobial therapy is still inconclusive and guidelines are yet to make a specific recommendation regarding combination therapy once susceptibility results are available. A retrospective study of 187 patients with *P. aeruginosa* BSI found that there was significant decrease in mortality in patients treated with definitive combination therapy compared to monotherapy by multivariate analysis (HR 0.30, 95% CI 0.13–0.71, *p* = 0.006) [1]. On the other hand, many studies have found no differences in outcomes between patients who received definitive combination vs. monotherapy [36]. For example, a retrospective study including 183 patients with *P. aeruginosa* VAP found similar outcomes with combination vs. monotherapy [95]. Furthermore, a meta-analysis found no difference in mortality between combination and monotherapy for patients with *P. aeruginosa* infections [37]. Hence, a single agent (preferably a beta-lactam based on susceptibility profile) should be used for definitive antimicrobial therapy since continuing combination therapy is unlikely to have any added value once susceptibilities are available [18].

## 4. Key Factors Related to Therapy

### 4.1. Antimicrobial Dosing

Due to the high level of intrinsic and acquired resistance among pseudomonal isolates, higher doses or extended infusions (EI) of beta-lactams may be necessary to ensure early attainment of target concentrations and maximize the duration of drug concentration required to exceed the MIC of the organism in severe infections [96]. The recommended doses for resistant and severe infections in the IDSA and ESCMID’s guidelines are higher than those used for other susceptible and mild infections [33,35]. In fact, in patients with severe sepsis or septic shock, the PK of most antibiotics are altered in the setting of an increased volume of distribution due to fluid administration and increased vascular permeability, altered renal clearance, and serum protein levels [97,98,99]. Hence, patients may require higher doses of antimicrobials to achieve efficient microbial killing [100]. For instance, a study using Monte Carlo simulation suggested that severe infections due to *P. aeruginosa* should preferably be treated with 2 g prolonged infusion of meropenem every 8 h rather than standard dosing (1 g every 8 h) [101]. Similarly, a PK/PD study reported that standard meropenem dosing may not be adequate for patients with non-susceptible organisms given that standard dosing did not achieve serum concentration over 2-times the MIC for over 40% of treatment duration in more than one-third of the patients. Instead, their PK modeling suggests that a higher dosage consisting of 500 mg bolus followed by 1500 mg extended infusion over 3 h every 8 h would achieve more adequate serum concentrations [102].

Another study aiming to optimize C/T dosing for the treatment of CRPA found that only the combination of C/T with amikacin as a loading dose of 20–25 mg/kg followed by 10–15 mg/kg/day achieved a cumulative fraction of response of >90% [103]. It should also be noted that critically ill patients who need renal replacement therapy may require higher dosing regimens to maintain effective serum concentrations [104]. In fact, although standard dosing of C/T of 1 g every 8 h achieves a serum concentration above the MIC for more than 40% of the treatment duration, a high dose of 2 g every 8 h might be needed to maintain a serum concentration above the MIC during the whole treatment duration [105]. In addition, the recommended dose of C/T for treatment of pneumonia is 3 g every 8 h based on the PK/PD modeling and according to which the ASPECT-NP trial dosing was based [73,106].

### 4.2. Infusion Rate

As previously discussed, the mainstay of treatment for *P. aeruginosa* sepsis are beta-lactams. However, beta-lactams exhibit a time-dependent effect on bacterial eradication and only achieve favorable microbiological and clinical outcomes when serum levels are maintained above the MIC during most of the duration of therapy. Prolonged infusion, whether given as extended infusion (EI) over multiple hours or as continuous infusions throughout the day, may help achieve a more sustainable serum concentration superior to the causative organism’s MIC. Despite all the challenges of EI, such as lack of intravenous access, tubing residuals, Y-site incompatibilities, and necessity for trained professionals, clinicians should opt for EI whenever possible to harvest its benefits. On many occasions, studies have shown that prolonged infusion may help improve patient outcomes. For instance, a retrospective cohort study including 194 patients with *P. aeruginosa* infections reported a 19.4% decrease in the 14-day mortality rate when comparing EI over four hours to standard intermittent infusion (*p*-value = 0.04). EI also shortened the duration of hospital stay by 17 days (*p*-value = 0.02) [107]. Another retrospective study including 87 patients with *P. aeruginosa* pneumonia and/or bacteremia who were treated with cefepime found that the overall mortality, length of stay in the ICU, and the need for ventilation were significantly lower in the EI group compared with the intermittent-infusion group [108]. According to a meta-analysis comparing EI to intermittent bolus (IB) (infusion over 0.5–1 h) in critically ill patients with severe *P. aeruginosa*, EI increases the probability of attaining serum concentrations superior to the causative organism’s susceptibility breakpoint, which is especially important for critically ill patients. In fact, using cefepime and piperacillin/tazobactam as EI consistently achieved concentrations above the breakpoints of susceptible agents only, but not concentrations above the breakpoints of resistant organisms. On the other hand, using EI for meropenem or doripenem achieved concentrations above the breakpoints for both susceptible and resistant organisms [109]. The accumulating evidence in favor of EI has lead both the IDSA and ESCMID to recommend its use for the treatment of non-susceptible strains [33].

On the other hand, the evidence regarding continuous infusion is still inconclusive. For example, a multicenter randomized controlled trial, the BLING II study, which included a total of 432 critically ill patients, showed no significant difference between intermittent and continuous infusion in ICU-free days, 90-day survival, duration of bacteremia, organ failure free days and clinical cure [110]. Additionally, 3 other meta-analyses have failed to show superiority of continuous infusion compared to IB [111,112,113]. Contrarily, the beta-lactam infusion in severe sepsis (BLISS) trial, which included a smaller population of 140 patients, found a higher clinical cure and fewer days on mechanical ventilation with continuous infusion [114] along with other clinical trials [115,116]. Thus, more randomized controlled trials are needed to draw definitive conclusions on the efficacy of continuous infusion of antimicrobial treatment for resistant *P. aeruginosa* sepsis.

### 4.3. Duration of Therapy

While many studies have supported the use of shorter antimicrobial courses to decrease AMR, cost, and adverse effects [117], the evidence on shortening the duration of treatment for a pseudomonal infection remains inconclusive. The duration of treatment for *P. aeruginosa* severe sepsis should be individualized according to the primary site of infection, the patient’s risk factors and underlying comorbidities, source control, susceptibility testing, trends of inflammatory biomarkers, and clinical improvement [118]. A recently published retrospective study comparing a short (6–10 days) course of antibiotics to a longer (11–15 days) course for *P. aeruginosa* bacteremia found no difference in mortality or bacteremia recurrence but found a significantly reduced hospitalization duration with shorter duration of treatment [119]. Moreover, a randomized controlled trial of 249 patients with *P. aeruginosa* BSI found no difference in mortality or recurrence when a course of 7 days was used compared to a course of 14 days and also reported shorter duration of hospitalization [120]. Hence, a shorter duration of treatment may be considered in immunocompetent patients who are showing clinical improvement and with a susceptible *P. aeruginosa*. However, a shorter duration may not be an option in immunocompromised patients like hematopoietic stem cell transplant (HSCT) patients who have a higher risk of recurrence if treated for less than 14 days [121]. For patients with sepsis secondary to pneumonia, we do not recommend a shortened treatment course of less than 14 days due to the high rate of recurrence in studies that compared short to long treatment durations [122,123].

## 5. Alternative Therapies

### 5.1. Phage Therapy

Phage therapy is a promising alternative therapies for patients who did not respond to conventional antibiotics [124]. Phages are viruses that can infect bacteria and are usually found in any natural environment where bacteria are present [125]. In clinical settings, phages can be used to target specific bacteria by migrating towards the site of infection, adhering to the cell surface of the targeted bacteria, and injecting their DNA into it. Phage therapy has the ability to significantly decrease bacterial loads, especially in *P. aeruginosa* biofilms, which is where antibiotics usually fail [126,127,128]. To date, there are over 700 phages infecting *P. aeruginosa* isolated and sequenced [129]. Several studies using phages against *P. aeruginosa* showed significant decrease in bacterial loads in vitro and ex vivo and improved survival rates in animals [126,130,131]. Three phages produced in Georgia are currently commercialized for use in *P. aeruginosa* sepsis. One clinical trial (NCT04636554) on personalized phage therapy in patients with COVID-19 and bacterial co-infection (including *P. aeruginosa* bacteremia/sepsis) is currently ongoing [132]. There are various case reports in humans showing clearance of *P. aeruginosa* using single or cocktail phage therapy in numerous infections, namely pressure ulcers with bacteremia [133], chronic wounds [134], chronic otitis [135], venous leg ulcers [136], and vascular graft infection [137]. Phage therapy was also combined with antibiotics (phage-antibiotic synergy) to increase bacterial killing of MDR *P. aeruginosa* in vitro [138,139]. There are several case reports of a combination of antibiotics with phage therapy to treat resistant *P. aeruginosa* infections, particularly in cases of chronic infections: antibiotics in various reports consisted of ceftazidime in endovascular infection with bacteremia [140]; meropenem, tobramycin, and polymyxin B in endovascular infection with bacteremia [141]; cefiderocol in cranial osteomyelitis [142], meropenem and colistin in UTI [143], CAZ/AVI in femur osteomyelitis [144], piperacillin/tazobactam, tobramycin, and colistin in lung transplant recipients [145], and other conventional antibiotics for empyema [146]. However, to date there is no data on the efficacy of phage therapy in sepsis.

### 5.2. Antibodies/Vaccines

Several vaccines targeting major components of *P. aeruginosa* have been developed to date, especially in patients with cystic fibrosis. These include vaccines against lipopolysaccharides, flagella, pili, type 3 secretion system, outer membrane proteins, and outer membrane vesicles as well as inactivated whole-cell [147]. The overwhelming majority of these vaccines have been focusing on eradicating or preventing lung infections, with few studies on *P. aeruginosa* bacteremia or sepsis [148]. Although vaccines have promising clinical applications, none have been marketed yet. A randomized clinical trial assessing the efficacy, safety, and immunogenicity of IC43 recombinant *P. aeruginosa* vaccine for mechanically ventilated ICU patients found that the vaccine achieved adequate immunogenicity but with no clinical benefits compared to placebo [149]. On the other hand, the PcrV protein, a part of the type three secretion system which allows the secretion of 4 exotoxins: U, S, T, and Y [150], has been associated with poorer clinical outcomes which has led to the development of monoclonal antibodies (mAb) against PcrV [151]. An anti-PcrV PE Gylated monoclonal antibody was assessed in a randomized double-blind controlled clinical trial and showed a favorable tolerance profile and a decreased *P. aeruginosa* pneumonia incidence in patients mechanically ventilated and colonized with *P. aeruginosa* [152]. This monoclonal antibody was also associated with improved survival when used in combination with antibiotics in mice [153]. Bispecific antibodies with a mAb targeting *P. aeruginosa* cross-linked with a mAb targeting the complement were tested in primates and showed a degree of protection against the bacterium [154]. Nevertheless, there are no clinical trials investigating the role of antibodies in the setting of *P. aeruginosa* sepsis and data might be extrapolated from studies on different sites of infection.

### 5.3. Quorum Sensing

*P. aeruginosa* uses quorum sensing, which is a signaling system implicating the exchange of chemical signals (or auto-inducers) within bacterial populations to regulate its phenotype and density. The concentration of these chemical signals can alter the gene expression of these bacteria by switching gene transcription on and off [155]. *P. aeruginosa* depends mainly on three interconnected quorum sensing systems (las, rhli, and the *Pseudomonas* quinolone signal (PQS)) which may be clinically relevant [156]. Several compounds have been shown to inhibit quorum sensing in *P. aeruginosa* including furanones, azithromycin, plant extracts, and garlic, in patients with cystic fibrosis who are chronically infected with *P. aeruginosa* [157,158,159,160].

### 5.4. Bacteriocins

Bacteriocins are peptides produced by bacteria that have a wide range of antimicrobial activity [161]. They are still in the early phase of assessment as potential alternatives to usual antimicrobial drugs, especially in catheter-associated UTI caused by *P. aeruginosa* [162,163].

## 6. Conclusions

The burden and mortality of *P. aeruginosa* severe sepsis is further exacerbated by the increased prevalence of resistant strains. Clinicians should have a high index of suspicion for pseudomonal sepsis in immunocompromised patients, those critically ill and patients with comorbidities and multiple hospitalizations. Treatment options for MDR and DTR strains are limited. Given the high mortality of severe sepsis due to *P. aeruginosa*, combination empirical treatment with two different mechanisms of action should be initiated without delay while waiting for the susceptibility results. However, de-escalation to monotherapy with an antimicrobial with the narrowest spectrum is highly advised once susceptibility is known. The introduction of the novel beta lactams has been a welcomed addition to the treatment armamentarium with good clinical efficacy and safety profile. The polymyxins are not recommended because of their significant nephrotoxicity and should only be used when no other options are available. Proper management leads to significant improvement in patient outcomes. Key factors including source control, EI, dosing adjustment, and appropriate treatment duration should be considered in the management of *P. aeruginosa* sepsis. With the advent of novel agents, emergence of resistance has been reported and the need for alternative therapies might be warranted. Several alternative treatments show early promising results but need to be tested in more rigorous studies. Applying stewardship principles in the management of patients is essential to ensure good outcomes and prevent the emergence of future resistance.

## Figures and Tables

**Table 1:** Treatment options for Carbapenem-resistant *P. aeruginosa* according to mechanism of resistance [67,68,69,70,71] C/T, Ceftolozane-Tazobactam; CAZ/AVI, Ceftazidime-avibactam; IMI/REL, Imipenem-cilastatin-relebactam.

	C/T	CAZ/AVI	IMI/REL	Cefiderocol	Pazomicin	Fosfomycin	Colistin
Carbapenemase							
Class A	No	Yes	Yes	Yes	Yes	Yes	Yes
Class B	No	No	No	Yes	Variable	Yes	Yes
Class D	No	Yes	No	Yes	Yes	Yes	Yes
OprD	Yes	Yes	Yes	Yes			
MexAB	Yes	No	Yes	Yes			
MexXY	Yes	No	Yes	Yes			

## Data Availability

Not applicable.
